# A case of mediastinitis with an exposed artificial blood vessel that was associated with right ventricular wall damage during treatment

**DOI:** 10.1080/23320885.2019.1611435

**Published:** 2019-05-10

**Authors:** Ryuichi Yoshida, Naoto Yamamoto, Akio Nishijima, Eri Maruyama, Megumi Takikawa, Satoshi Yanagibayashi

**Affiliations:** a Department of Plastic and Reconstructive Surgery, New Tokyo Hospital, Chiba, Japan;; b Department of Plastic and Reconstructive Surgery, University of Jichi Medical University Saitama Medical Center, Saitama, Japan

**Keywords:** Mediastinitis, right ventricle wall rupture, exposed artificial vessel, Negative Pressure Wound Therapy with continuous instillation(NPWT-CI), pectoralis major muscle flap

## Abstract

We report a serious case of right ventricular wall damage during mediastinitis treatment, which was successfully treated with negative-pressure wound therapy with continuous instillation (NPWT-CI).

## Introduction

Mediastinitis after cardiotomy has been reported to occur in 1%–3% of cases, and its mortality rate has been reported to be 19%–29% [1]. Mediastinitis is a serious complication, and its treatment becomes difficult when an artificial blood vessel is exposed.

Also, mediastinitis treatment causes right ventricular wall damage in approximately 0.8%–14.6% of cases, and a high rate of death from massive bleeding has been reported [2].

Herein, we report a rare case of mediastinitis with an exposed artificial blood vessel that was associated with ventricular wall damage during treatment.

The patient was successfully treated using negative-pressure wound therapy with continuous instillation (NPWT-CI).

## Case

### Patient details

The patient was a 74-year-old man with the chief complaint of precordial erythema.

He had a history of hypertension, hyperlipidaemia, and appendicitis.

### Clinical history

He had developed aortic dissection of Stanford type B in August 2012.

He was followed up conservatively in the outpatient department; however, the dissection diameter gradually increased. He was introduced to our cardiovascular department for surgery in July 2016.

Hemiarch replacement and open stent grafting were performed by a cardiac surgeon in August 2016.

After surgery, he was admitted to the intensive care unit and was extubated on the 4th day. Erythema developed at the precordial operation scar on the 24th day after surgery, and thus, he was introduced to our plastic surgery department.

### Clinical findings

**Figure 1.**
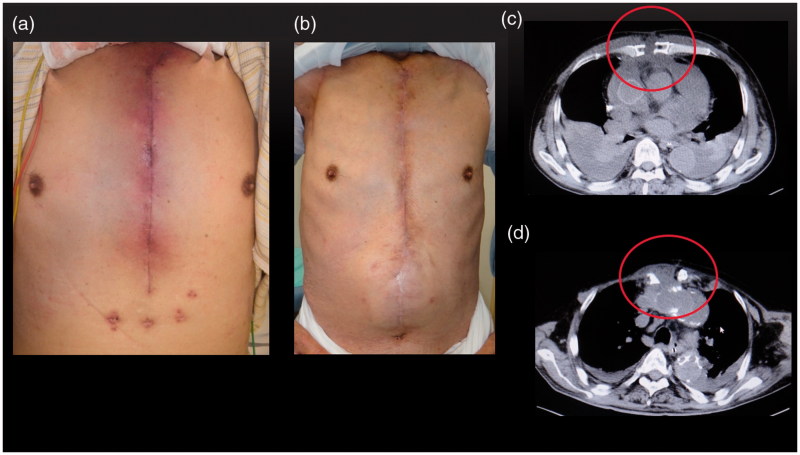
(a) Clinical observation: erythema at the precordial operation scar. (b) Six months after surgery: no recurrence. (c) Preoperative computed tomography: sternal ablation and liquid retention. (d) Postoperative computed tomography: dead space filled with a pectoralis major muscle flap.

Erythema was noted at the precordial operation scar (Figure 1a).

His blood test results were as follows: white blood cell count, 6370/µL; haemoglobin level, 0.2 g/dL; platelet count, 20.3 × 10^4^/µL; aspartate aminotransferase/alanine aminotransferase level, 29/36 U/L; blood urea nitrogen/creatinine level, 24.3/0.77 mg/dL; TP level, 7.4 g/dL, albumin level, 2.5 g/dL; and C-reactive protein level, 5.65 mg/dL. On computed tomography, ablation of the sternum and liquid retention at the sternum were noted (Figure 1c).

### Clinical course

Debridement was performed under general anaesthesia 3 days after introduction to our department. All wires, a sequestrum, and sphacelus were removed surgically, resulting in the exposure of the mediastinum and artificial blood vessel

As the infection sign of the wound was strong, we washed the wound with a large amount of saline and left it open. After the operation, we covered the wound with a large amount of gauze and fixed the chest with a breast harness.

On the day following the operation, after the patient rose to the sitting position, a large amount of bleeding was noted at the gauze, and his systolic pressure fell to 80 mmHg. He was transferred to the operating room with rapid blood transfusion, and an emergency operation was performed for hemostasis. We found that the outer layer of the right ventricular wall had split, and arterial bleeding was seen.

**Figure 2.**
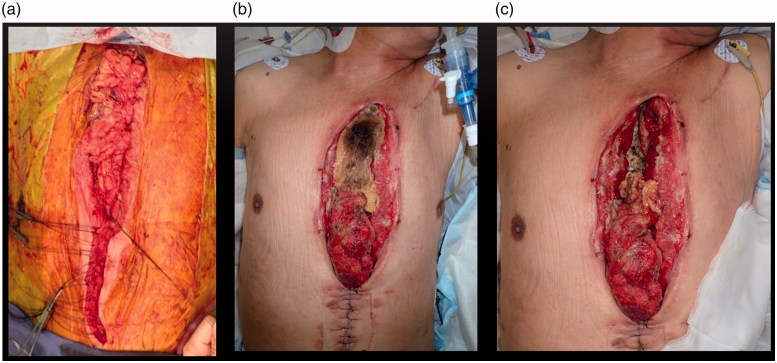
(a) Image during greater omentum filling. (b) Image obtained on the 14th postoperative day: cranial half of the greater omentum shows necrosis. (c) Imege after necrotic greater omentum removal: artificial blood vessel is exposed.

Bleeding was stopped with a surgical clip, the mediastinal space was filled with the greater omentum, and the wound was closed (Figure 2a). After the operation, he was admitted to the intensive care unit and was placed on a respirator.

On the day following the operation, bleeding again occurred after coughing owing to sputum suction, and his systolic pressure decreased to 50 mmHg.

He was transferred to the operating room with rapid blood transfusion, and an emergency operation was again performed for hemostasis. We found that a new laceration on the right ventricular wall had penetrated into the right ventricular cavity. As the damaged wall was weak, simple suturing could not be performed. Thus, patch closure was performed with his pericardium.

After the bleeding stopped, the mediastinal space was again filled with the greater omentum; however, the wound could not be closed because of swelling. Thus, the wound was covered with a Gore-Tex sheet. The patient received 20 units of red blood cells, 24 units of fresh frozen plasma, and 20 units of platelet concentrate in total. The wire and sequestrum obtained during debridement were cultured; however, bacteria were not detected.

**Figure 3.**
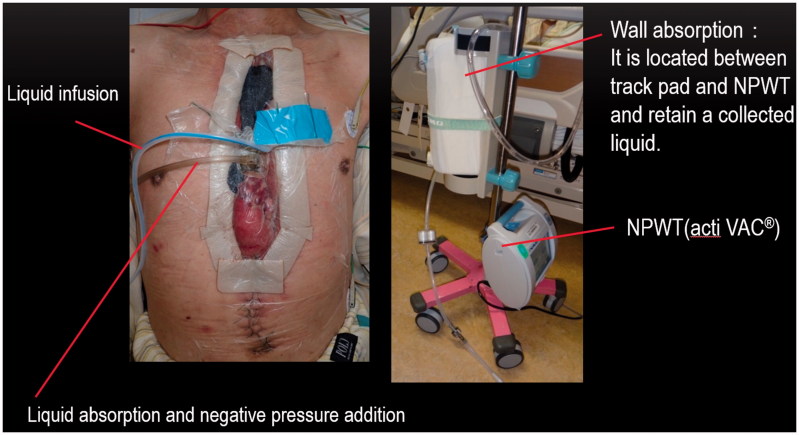
Negative-pressure wound therapy with continuous instillation (NPWT-CI).

The upper half of the greater omentum showed necrosis after the second hemostasis operation, and we removed the necrotic part surgically on the 14th day after the operation (Figure 2b). After removal, the artificial blood vessel was greatly exposed (Figure 2c). We later washed the wound with saline (500–1000 mLwashing) and covered the wound with iodoform dressing or silver dressing twice a day. However, the infection sign did not disappear. Thus, we performed NPWT-CI according to the method reported previously (Figure 3) [3]. NPWT-CI was performed for 31 days with a pressure setting of 75–100 mmHg and saline use of 2 L/day. The infection sign gradually reduced, and wound closure was planned as the back of the artificial blood vessel appeared buried with granulation tissue. During NPWT-CI, bacteria were not detected three times.

**Figure 4.**
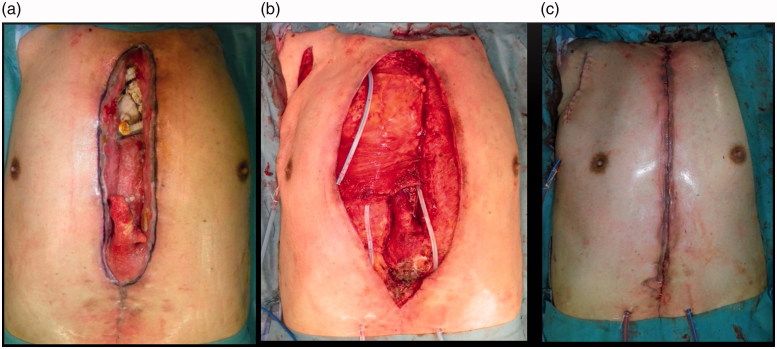
Reconstructive surgery. (a) Preoperative view. (b) Intraoperative view: dead space is filled with a right pectoralis major muscle flap. (c) Image after surgery.

After debridement, the right pectoralis major muscle was separated with a humeral adhesion portion and was turned over to fill the cavity around the artificial blood vessel. The dead space was filled with the greater omentum, which remained in the caudalis, and a left pectoralis major advancement muscle flap was used to close the wound (Figure 4). He visited our hospital on foot for 6 months after the operation and did not show recurrence (Figure 1b, d).

## Discussion

A previous report of 42 cases of right ventricular wall damage during mediastinitis treatment mentioned that the occurrence rate of right ventricular wall damage was 0.8%–14.6%, coronary artery bypass grafting was the primary method of cardiac operation, and the mean onset time from mediastinitis operation to right ventricular wall damage was 2.9 days (range, 30 min–12 days) [2].

As the damaged right ventricular wall is often fragile, simple closure can be difficult. Thus, patch closure with self-pericardium is often performed. The utilisation rate of the heart-lung machine has been reported to be 62%, and the death rate has been reported to be 20% [2]. The following causes of right ventricular wall damage have been proposed: shearing force due to coughing, pressure on the sternal wall associated with the right ventricular wall during NPWT, and removal of the adhered foam material during NPWT [2]. In the present case, we believe that right ventricular wall damage occurred from shearing force, as massive bleeding was noted after coughing. To prevent damage from shearing force, it is important to avoid adhesion of the right ventricular wall and the back of the sternum [2].

Previously, the wound was commonly closed with an omental flap after debridement for mediastinitis therapy. However, the incidence of two-stage closure has increased because of the development of NPWT. The present patient recovered from his critical condition and left the hospital alone on foot. We believe that he survived because of quick hemostasis achievement, excellent wound bed preparation by NPWT-CI, and second closure with a pectoralis major muscle flap.

NPWT is reportedly useful as a bridge from debridement to second closure for mediastinitis [4–6]. However, when the infection sign is strong, careful attention is needed. As occlusion treatment is involved, the number of bacteria might increase [7]. Infection control is complicated when an artificial blood vessel is exposed. For such cases, the usefulness of NPWT with washing has been highlighted. Svedman® [8], IW-conpit [9], and a novel approach [3] have been described for NPWT-CI. VAC Ulta^®^ has been shown for NPWT with instillation (NPWT-I). We could not use VAC Ulta^®^ as it was introduced in Japan in August 2017. IW-conpit or existing NPWT, which involves wall absorption for continuous irrigation, has frequently been used in Japan. In the present case, we performed NPWT-CI according to the method reported by Sakakibara et al. [3] This method increases negative pressure at the wound while the wound is continuously perfused with saline at 2–3 L/day. The quantity of saline can be increased or decreased depending on the situation, and washing of the wound with a large amount of saline continuously is beneficial.

There is controversy as to which of antiseptic solution and normal saline is superior.

According to only RCT [10] which compared normal saline with antiseptic solution (0.1％polyhexanide plus 0.1% betaine), it is reported that normal saline might be as effective as an antiseptic.

The possibility of a “shunt” has been indicated as a disadvantage of NPWT-CI. Thus, NPWT-CI may not be able to appropriately wash every part of the wound when compared with NPWT-I. It has been reported that the number of bacteria can be better reduced with NPWT-I than with normal NPWT [11,12]; however, it is not known whether NPWT-CI or NPWT-I is more beneficial in infection control. Thus, studies comparing the infection control abilities of NPWT-CI and NPWT-I are required.

## Conclusion

We reported a serious case of right ventricular wall damage during mediastinitis treatment after aortic arch replacement, which was successfully treated with NPWT-CI. We believe that urgent operation for hemostasis and good wound bed preparation by NPWT-CI helped achieve a good outcome.
